# AFN-Net: Adaptive Fusion Nucleus Segmentation Network Based on Multi-Level U-Net

**DOI:** 10.3390/s25020300

**Published:** 2025-01-07

**Authors:** Ming Zhao, Yimin Yang, Bingxue Zhou, Quan Wang, Fu Li

**Affiliations:** 1 School of Computer Science, Yangtze University, Jingzhou 434025, China; hitmzhao@gmail.com (M.Z.); yangyimin187@gmail.com (Y.Y.); 2 School of Internet of Things Engineering, Wuxi University, Wuxi 214105, China; seepwq@163.com (Q.W.); lifu@cwxu.edu.cn (F.L.)

**Keywords:** U-Net, medical image segmentation, small target segmentation, feature fusion, loss function

## Abstract

The task of nucleus segmentation plays an important role in medical image analysis. However, due to the challenge of detecting small targets and complex boundaries in datasets, traditional methods often fail to achieve satisfactory results. Therefore, a novel nucleus segmentation method based on the U-Net architecture is proposed to overcome this issue. Firstly, we introduce a Weighted Feature Enhancement Unit (WFEU) in the encoder decoder fusion stage of U-Net. By assigning learnable weights to different feature maps, the network can adaptively enhance key features and suppress irrelevant or secondary features, thus maintaining high-precision segmentation performance in complex backgrounds. In addition, to further improve the performance of the network under different resolution features, we designed a Double-Stage Channel Optimization Module (DSCOM) in the first two layers of the model. This DSCOM effectively preserves high-resolution information and improves the segmentation accuracy of small targets and boundary regions through multi-level convolution operations and channel optimization. Finally, we proposed an Adaptive Fusion Loss Module (AFLM) that effectively balances different lossy targets by dynamically adjusting weights, thereby further improving the model’s performance in segmentation region consistency and boundary accuracy while maintaining classification accuracy. The experimental results on 2018 Data Science Bowl demonstrate that, compared to state-of-the-art segmentation models, our method shows significant advantages in multiple key metrics. Specifically, our model achieved an IOU score of 0.8660 and a Dice score of 0.9216, with a model parameter size of only 7.81 M. These results illustrate that the method proposed in this paper not only excels in the segmentation of complex shapes and small targets but also significantly enhances overall performance at lower computational costs. This research offers new insights and references for model design in future medical image segmentation tasks.

## 1. Introduction

Medical image segmentation, as one of the core tasks of computer vision in the medical field, has made significant progress in several subfields, playing a vital role in applications such as organ recognition, lesion detection, and surgical planning [1]. Over the past decade, deep learning-based models have greatly advanced the field of medical image analysis, particularly with the widespread application of convolutional neural networks (CNNs). Models like U-Net have achieved tremendous success in biomedical image segmentation. U-Net [2], with its symmetrical encoder–decoder architecture, enables the fusion of multi-scale information and has been widely applied to segmentation tasks such as brain tumors, cardiac structures, and pulmonary nodules [3,4,5].

In recent years, with the increase in diversity and complexity of medical images, researchers have proposed a variety of improved segmentation network structures based on U-Net. The nnU-Net [3] achieves excellent performance in a wide range of medical image segmentation tasks, especially in brain tumor segmentation, through adaptive network configuration. In addition, 3D-network models such as NestedFormer, Transbts [6,7,8] are widely used in medical image segmentation, such as the 3D segmentation of brain and heart, which enhances the capture of spatial features. The ds-transunet and other models [9,10,11] are based on the attention mechanism and multi-scale feature fusion, which enhances the segmentation performance of complex anatomical structures, especially in the significant effect when dealing with multi-scale information. However, these networks still have some limitations in segmentation tasks with complex boundaries and small targets.

To further improve segmentation performance, researchers have developed models that combine Transformer [12,13] with classic architectures. For instance, Trans U-Net [14], which integrates Transformer with the U-Net architecture, leverages Transformer’s strong capability to capture long-range dependencies, achieving remarkable results in large-target segmentation tasks such as cardiac and multi-organ segmentation tasks. Similarly, the SWIN U-Net [15] combines the strengths of both Transformer and CNN, effectively improving segmentation accuracy in multi-organ segmentation. Furthermore, the DeepLab series has also incorporated Transformer structures by introducing self-attention mechanisms to enhance multi-scale information capture. TransDeepLab [16], for example, has demonstrated outstanding performance in skin lesion and multi-organ segmentation tasks.

Although models combining Transformer and CNN have achieved good results in large-target segmentation tasks, they still face significant challenges when dealing with small-target segmentation [17]. While these models can capture global information and long-range dependencies, the subtle features of small targets are often overshadowed by global information, leading to insufficient focus on small targets. Additionally, small targets are often characterized by complex shapes, blurred boundaries, and low contrast with the background, further increasing the difficulty of segmentation [18]. For instance, in nucleus segmentation tasks, overlapping nuclei and unclear boundaries make it difficult for traditional image processing methods to accurately identify these small targets, thereby affecting the overall results [19,20].

To address these challenges, researchers have proposed several deep learning-based nucleus segmentation methods. He et al. [21] proposed an improved U-Net architecture that integrates multi-scale feature fusion and boundary enhancement to improve the accuracy of the segmentation. Additionally, in recent years, attention mechanism-based models [22,23] have significantly improved accuracy by enhancing the focus on key regions. Some methods have also combined CNNs with Transformer architectures, which excels in nucleus segmentation by leveraging the strengths of both Transformers and CNNs [24,25,26]. However, these models still face challenges when dealing with complex backgrounds and highly overlapping nuclei, such as blurry boundaries and indistinct features [24,25,26,27,28].

To solve these problems, this paper proposes a new nucleus segmentation method based on the U-Net architecture with three modules: the WFEU, the DSCOM and the AFLM. Through the synergy of these modules, the proposed method not only improves the accuracy and robustness of the model in handling complex nucleus segmentation tasks but also demonstrates advantages in computational efficiency. First, WFEU enhances the model’s feature representation ability by adaptively assigning feature weights, allowing for more precise capture and highlighting of key features in complex backgrounds. Second, DSCOM optimizes channel information through multi-level convolution operations, ensuring high-resolution features are retained in small-target and boundary regions, thus significantly improving segmentation accuracy. Finally, AFLM dynamically adjusts the weights of various components in the loss function, effectively balancing the requirements for classification accuracy, region consistency, and boundary precision, thereby improving the model’s overall performance. Experimental results show that the proposed segmentation method performs excellently when dealing with complex shapes and highly overlapping nuclei. This method not only improves the accuracy of nucleus segmentation but also demonstrates high computational efficiency and stability, providing new insights and methods for future research in the field of small-target segmentation.

The structure of this paper is organized as follows: Section 2 reviews the related research work; Section 3 provides a detailed description of the proposed method; Section 4 shows the experimental results and analysis; Section 5 conducts the ablation experiments to validate the validity of the modules; and Section 6 concludes the study of this paper.

## 2. Related Work

### 2.1. Feature Fusion

**Figure 1:**
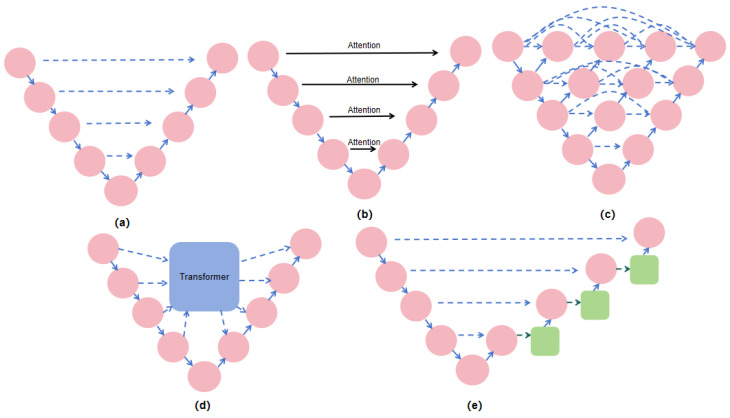
Structure of skip connection methods: (**a**) U-Net structure, (**b**) Attention U-Net structure, (**c**) U-Net++ structure, (**d**) Transformer-based structure, (**e**) structure proposed in this study.

U-Net [2] and its derived architectures have a wide range of applications and research backgrounds in medical image segmentation tasks. As a classical encoder–decoder architecture, the uniqueness of U-Net lies in its hopping connections, which connect the feature maps between the encoder and the decoder directly, resulting in better preservation of the high-resolution information during decoding, and consequently improving the segmentation accuracy. In the original U-Net architecture, feature maps were fused through simple concatenation. While this direct fusion is effective, it may introduce certain limitations when dealing with complex backgrounds or fine details, as it lacks selective enhancement of key features. To address this issue, subsequent research has proposed various improvements. Figure 1 illustrates different types of skip connection structures.

By introducing dense skip connections, U-Net++ [29] makes the feature maps of different resolutions fuse step by step, reduces the semantic gap and improves the segmentation performance. Attention U-Net [30] further incorporated attention mechanisms at the skip connections, allowing the network to selectively enhance key feature regions while suppressing irrelevant background information, resulting in higher robustness and accuracy in complex segmentation tasks. To meet the demand for lightweight models, the UNeXt [31] network adopted an efficient feature fusion strategy between the encoder and decoder, significantly reducing computational costs while maintaining excellent segmentation performance. UNeXt incorporates depthwise separable convolutions and efficient feature extraction modules, enabling high-efficiency medical image segmentation even in resource-constrained environments. Building on this, DCA [32] introduced a dual-channel attention mechanism, further enhancing feature selectivity and allowing for more precise focus on target regions, thereby improving segmentation accuracy. M2SNet [33] incorporated a multi-scale selection mechanism during feature fusion, enabling the network to better capture target information at different scales.

Building upon the U-Net architecture, this study further optimizes the feature fusion between the encoder and decoder, thereby improving the model’s adaptability and accuracy in complex medical image segmentation and enhancing its performance in diverse medical image segmentation tasks.

### 2.2. Deep Convolution

Convolution operations are the core components of CNNs that extract features from images through local receptive fields. With the continuous advancement of deep learning technologies, researchers have proposed various innovative convolution operations to improve computational efficiency and feature extraction capabilities, meeting the diverse needs of different applications.

Depthwise separable convolution, as an important optimization of convolution operations, has garnered widespread attention and application in recent years. First introduced by Chollet in the Xception model [34], it decomposes standard convolution into depthwise convolution and pointwise convolution, which significantly reduces computational complexity and the number of parameters. This method preserves efficient feature extraction while greatly reducing the consumption of computational resources.

The efficiency of depthwise separable convolution has made it widely adopted in various lightweight network architectures, such as MobileNetV3 [35] and EfficientNetV2 [36]. These models optimize convolution operations to further enhance performance in resource-constrained environments. At the same time, the application of depthwise separable convolution in medical image segmentation has also been validated. For example, Magadza et al. [37] used this convolution method in their brain tumor segmentation network, significantly improving inference speed and accuracy, making it suitable for real-time clinical applications. Similarly, Dutande et al. [38] applied depthwise separable convolution to the automatic detection and segmentation of lung nodules, dramatically reducing computational costs while maintaining high segmentation accuracy.

In this study, our proposed WFEU and DSCOM extensively draw on the advantages of deep convolution. By introducing deep convolution, these modules effectively reduce the computational complexity while ensuring the accuracy of the model, demonstrating the important role of deep convolution in optimizing the network structure.

## 3. Method

### 3.1. Network Design

**Figure 2:**
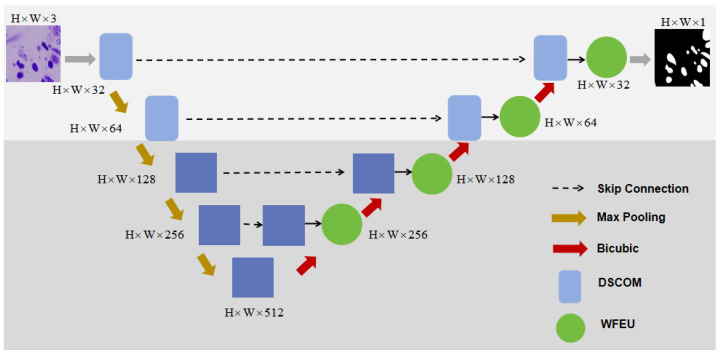
Overall structure of the model. The overall framework is a five-layer structure. The first two layers are convolutional structures, and the last three layers are Transformer structures. WFEU is used to fuse the features of the encoder and decoder.

To address the challenges of complexity and diversity in the nucleus segmentation task, we propose an improved U-Net architecture. The overall network structure is shown in Figure 2. This network retains the standard U-Net encoder and decoder components while introducing several key modules, including WFEU, DSCOM, and AFLM. The introduction of these modules enables multi-scale feature extraction, feature fusion, and optimization of boundary segmentation. The design and implementation of each key module will be described in detail.

### 3.2. Weighted Feature Enhancement Unit (WFEU)

**Figure 3:**
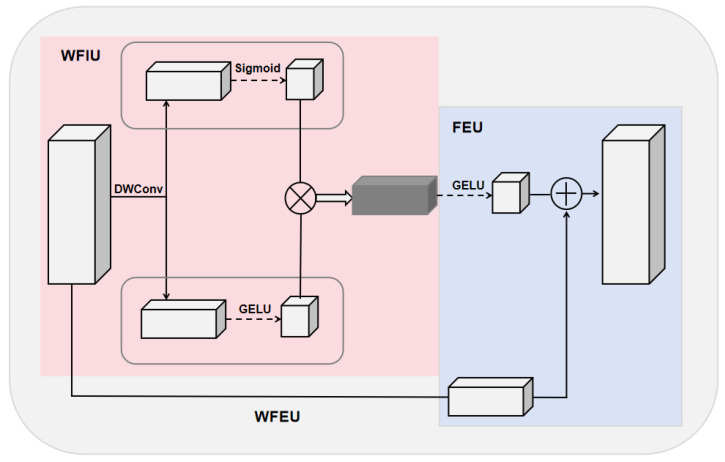
Structure of the WFEU module. WFEU consists of two main small units. The pink area on the left side is WFIU, and the blue area on the right side is FEU.

In the task of nucleus segmentation, the model needs to accurately capture complex nucleus boundaries and internal structures in high-resolution images. To achieve this, we designed the WFEU. With this design, we effectively enhance and fuse input features, enabling the model to better capture complex boundaries and internal structures, thereby improving the overall performance of the task. The WFEU consists of two main subunits: a Weighted Feature Interaction Unit (WFIU) and a Feature Enhancement Unit (FEU), as shown in Figure 3.

First, the low-resolution semantic features generated by the encoder and the high-resolution spatial features produced by the decoder are concatenated to form a feature map containing multi-scale information. This concatenated feature map serves as the input to the WFEU. Then, the input feature map is first processed by the WFIU, generating interactive features. These interactive features capture important parts of the input features, reducing the influence of background noise. Next, the interactive features are passed into the FEU, where convolution operations at different scales improve the model’s ability to capture both global characteristics and local details of the nucleus, achieving effective multi-level feature fusion.

#### 3.2.1. Weighted Feature Interaction Unit (WFIU)

The design of the WFIU aims to selectively enhance the important parts of the input features through dynamic weighting and nonlinear transformations, thereby improving the precision of feature representation. First, the WFIU generates a dynamic weight map to control the weight distribution across different regions, highlighting salient features. Specifically, the input feature map *x* generates dynamic weights by $x_{1}$:

(1) $$x_{1}=\sigma \left(W_{1}*x+b_{1}\right)$$

Next, the input features are transformed by a nonlinear transformation to generate a more expressive feature, $x_{2}$:

The formula is as follows:

(2) $$x_{2}=GELU\left(W_{2}*x+b_{2}\right)$$

Ultimately, the dynamic weight map interacts with the nonlinear features to produce an interaction feature, $x_{3}$:

(3) $$x_{3}=x_{1}*x_{2}$$

where $W_{1}$ and $W_{2}$ represent convolution kernels, $b_{1}$ and $b_{2}$ represent bias terms, and σ and *GELU* represent activation functions.

#### 3.2.2. Feature Enhancement Unit (FEU)

The FEU is designed to fuse feature information from different sources through further processing and channel alignment operations. First, the interaction feature $x_{3}$ further enhances its representation by nonlinear transformations to generate an enhanced feature, $x_{4}$:

(4) $$x_{4}=GELU\left(W_{3}*x_{3}+b_{3}\right)$$

Subsequently, the input feature map x is passed through the 1 × 1 convolution to adjust the number of channels to ensure alignment with the enhancement features, generating the aligned feature $x_{5}$:

(5) $$x_{5}=W_{4}*x+b_{4}$$

Based on this, the enhanced features are summed with the aligned input features to form the final output feature map out:

(6) $$out=x_{4}+x_{5}$$

where $W_{3}$ represents the convolution operation, $W_{4}$ represents the 1 × 1 convolution operation, $b_{3}$ and $b_{4}$ represent the bias term, and *GELU* denotes the activation function. FEU significantly improves the representation of the feature map by combining multi-level feature information, enabling the model to capture more complex patterns and structures.

### 3.3. Double-Stage Channel Optimization Module (DSCOM)

In the segmentation task, the diverse shapes and blurred boundaries of nuclei make it difficult for traditional convolutional neural networks to simultaneously capture both feature extraction and computational efficiency. To solve this problem, we propose DSCOM, which is applied to the first two layers of the whole framework to extract the high-frequency global features of the original input image. The high-frequency features contain color, texture, shape and other information, but there are problems such as high feature dimensions and large amounts of calculation. In this paper, by combining the advantages of ordinary convolution and deep convolution, more comprehensive feature extraction and optimization are realized, and the problems of calculation parameters and complexity are solved.

A two-stage design is adopted in the DSCOM module. The first stage performs a standard convolution operation, transforming the number of input feature channels from $C_{in}$ to $C_{out}$, thereby capturing more global features at an early stage. This transformation enables the network to better adapt to complex images. Next, we introduce depthwise convolution, where the convolution is applied independently to each channel while keeping the number of channels unchanged. This design effectively reduces the number of parameters and computational cost, while preserving spatial information within each channel, which is particularly useful for precise nucleus boundary information extraction. Finally, we utilize batch normalization and ReLU activation functions in the first stage to regularize the output and introduce non-linear features to further enhance the hierarchy of feature expression and the robustness of the model.

The second stage repeats a similar sequence of operations as the first stage, but the input is the output feature map from the first stage. Through further convolution and optimization of the first stage’s output, DSCOM is able to maintain feature complexity while enhancing the network’s feature extraction capability. This design not only helps the model to accurately capture subtle variations in nucleus morphology, but also effectively reduces interference from background noise to improve segmentation accuracy.

Mathematically, the operation of the entire DSCOM module can be represented as a two-stage composite function:

(7) $$f\left(X\right)=g_{2}\left(g_{1}\left(X\right)\right)$$

The first stage $g_{1}\left(X\right)$ is specified as:

(8) $$g_{1}\left(X\right)=\sigma \left(B_{C_{out}}\left(D_{C_{out}}\left(C_{C_{in}\to C_{out}}\left(X;W_{1}\right)\right);W_{2}\right)\right)$$

Phase II $g_{2}\left(Z_{1}\right)$ can be specified as:

(9) $$g_{2}\left(Z_{1}\right)=\sigma \left(B_{C_{out}}\left(D_{C_{out}}\left(C_{C_{out}\to C_{out}}\left(Z_{1};W_{3}\right)\right);W_{4}\right)\right)$$

where *X* is the input feature map tensor with the size of $C_{in}\times H\times W$, and $Z_{1}$ is the output tensor of the first stage with the size of $C_{out}\times H\times W$. $C_{in}$ and $C_{out}$ are the number of input and output channels, respectively, and *H* and *W* are the height and width of the feature map, respectively. $W_{1},W_{2},W_{3}$ and $W_{4}$ represent the convolution kernel weight tensors. $C\left(X;W\right)$ denotes the normal convolution operation; $D\left(X;W\right)$ denotes the deep convolution operation; $B\left(Y\right)$ denotes the batch normalization operation; and $\sigma \left(Z\right)$ is the ReLU activation function.

### 3.4. Adaptive Fusion Loss Module (AFLM)

One of the main challenges in nucleus segmentation tasks lies in its high imbalance, where the nucleus regions are typically much smaller compared to the background, and the boundaries of the nuclei are often blurry and in complex shapes. Traditional single loss functions often struggle to balance global classification accuracy with local boundary details, resulting in poor performance in small-target segmentation and boundary precision. To enhance the performance of the model, we designed a new comprehensive loss function, AFLM, which integrates BCE, Dice, and boundary losses to optimize the model’s performance in pixel-level classification, regional consistency, and boundary precision.

To further optimize boundary segmentation accuracy, we introduced a boundary loss in the loss function. The boundary loss measures the discrepancy between the predicted boundaries and the ground truth boundaries, ensuring that the model can still accurately segment complex and blurred nucleus boundaries. Specifically, the boundary loss relies on the following two parts of the calculation.

First, define x∈Ω, which means that the boundary point *x* belongs to the image domain Ω; i.e., it is a pixel point in the image. $\Omega \subseteq R^{2}$ denotes the image domain, which is usually the pixel plane of the image. δ and ϵ are threshold parameters defining the boundary, which are used to indicate whether the label changes within a small range of distances, and the region where the label changes is recognized as the boundary. δ>0 denotes a positive number that is used to define a domain range. ∀ϵ<δ denotes all possible domain pixels within this very small range. *G* denotes the labeling of the true segmentation result, and $G\left(x\right)$ denotes the true labeling on position *x*. *P* denotes the model-predicted segmentation result, and $P\left(x\right)$ denotes the predicted label on position *x*. For binary segmentation issues, usually 0 denotes the background and 1 denotes the foreground. $G\left(x+\epsilon \right)\neq G\left(x\right)$ and $P\left(x+\epsilon \right)\neq P\left(x\right)$ are boundary conditions that denote the change in the value of the true label *G* or the predicted label *P*, i.e., from foreground to background or from background to foreground, in a very small domain of pixel point *x*, which implies the existence of a boundary at point *x*. The following formulas define the true segmentation boundaries and predicted boundaries that help the model to optimize the boundary detection of the target in the segmentation task.

(10) $$B\left(G\right)=\{x\in \Omega \;\left|\;\exists \delta >0,\forall \epsilon <\delta ,G\left(x+\epsilon \right)\neq G\left(x\right)\right.\}$$

(11) $$B\left(P\right)=\{x\in \Omega \;\left|\;\exists \delta >0,\forall \epsilon <\delta ,P\left(x+\epsilon \right)\neq P\left(x\right)\right.\}$$

$B\left(G\right)$ denotes the true boundary, which defines the set of all points at which the true label $G\left(x\right)$ changes; i.e., in the minimal domain ϵ of *x*, *x* is considered to be a boundary point if $G\left(x+\epsilon \right)$ is not equal to $G\left(x\right)$, which represents the boundary of an object or foreground target. Similarly, $B\left(P\right)$ denotes the prediction boundary. The prediction label $P\left(x\right)$ of the set of boundary points defines the points in the predicted segmentation where the label change occurs, which represents the object boundary predicted by the model. By strictly defining the boundaries, the formulas can optimize the model by further boundary loss and make the model perform better in segmentation tasks with complex boundaries.

Then, by solving for the minimum distances between the pixel points and the real boundary, the performance of the segmentation model in the boundary region will be improved by reducing these distances.

(12) DGx=∥x−y∥2y∈BGinf

where $D_{G\left(x\right)}$ denotes the distance from point x to the true boundary $B\left(G\right),$ which is the shortest distance from the target point *x* and is used to measure the distance from the pixel point to the true segmentation boundary in the prediction result. $y\in B\left(G\right)$ denotes that point y belongs to the real boundary, and ${∥x-y∥}_{2}$ is the Euclidean distance, which is the straight-line distance between *x* and *y*; i.e., the distances between the predicted point *x* and all the points *y* on the boundary are calculated, and the shortest distance among them is selected. inf is the sign for finding the minimum value. $D_{G\left(x\right)}$ represents the distance from point *x* to the nearest boundary point. By optimizing the distance of boundary points, the model can effectively reduce the error in the fuzzy boundary area, making it possible to better deal with complex morphology of the nuclei and highly overlapping nucleus regions, and ensuring that the model can better capture the accuracy of the boundary.

At the same time, we compute the Euclidean paradigm of the gradient, which is used as a measure of how much the point *x* is predicted to be in the result $P\left(x\right)$. That gradient is a measure of the change in image intensity, which is used to reflect the extent to which the image changes around point *x*. $\frac{B\left(P\right)}{\partial x}$ and $\frac{B\left(P\right)}{\partial y}$ represent the partial derivatives of *p* with respect to *x* and *y*, respectively, indicating the rate of change of the image in the horizontal and vertical directions. $\nabla P\left(x\right)$ denotes the gradient of the function *P*(*x*), $and\;{∥\nabla P\left(x\right)∥}_{2}$ denotes the L2 paradigm of the gradient, which is used to measure the magnitude of the change in the image by calculating the square root of the sum of the squares of the gradient in different directions. In an image, places with large gradients often correspond to edges in the image, and these edges are usually the boundaries of the target object. Therefore, the gradient paradigm can help the model to focus on the edge regions and improve the recognition of the boundaries. For tasks such as nucleus segmentation, accurate identification and segmentation of boundaries are critical to model performance.

(13) $${∥\nabla P\left(x\right)∥}_{2}=\sqrt{{\left(\frac{B\left(P\right)}{\partial x}\right)}^{2}+{\left(\frac{B\left(P\right)}{\partial y}\right)}^{2}}$$

Finally, the boundary loss is defined as $L_{Boundary\left(P,G\right)}$. Through this loss function, the similarity between the prediction result *P* and the real boundary *G* can be strengthened, effectively avoiding the problem of predicting the segmentation boundary blurring or deviating from the real boundary. Especially in the nucleus segmentation task, the boundary is often fuzzy, and by optimizing the boundary loss, the model can better deal with fine and complex boundary problems. The formulation combines the gradient paradigm with the boundary distance to help the model more accurately localize and capture the boundary information in the image. The *dx* denotes the integral variable, which represents the accumulation for each pixel point *x* in the image.$\int_{\Omega }{∥\nabla P\left(x\right)∥}_{2}\cdot D_{G\left(x\right)}dx$ denotes the entire image domain Ω. Integration is performed. The integration operation is used to accumulate the gradient and distance values over the entire image to get the overall boundary loss.

(14) $$L_{Boundary\left(P,G\right)}=\int_{\Omega }{∥\nabla P\left(x\right)∥}_{2}\cdot D_{G\left(x\right)}dx$$

In the design of AFLM, the $BCE\left(P,G\right)$ denotes the binary cross-entropy loss function, which is used to measure the difference between the model’s predictions and the true labels, and which guides the model to gradually learn more accurate segmentation results by imposing a larger penalty on incorrect predictions. Ω denotes the pixel set of the entire image, which is the indexed set of all pixels in the image. |Ω| denotes the total number of pixels in the image. This term is a normalization factor that ensures that the loss function affects all pixels evenly. $\sigma \left(P\left(x\right)\right)$ is the output of the sigmoid activation function in the range [0, 1], and $log\left(\sigma \left(P\left(x\right)\right)\right)$ and $\log\left(1-\sigma \left(P\left(x\right)\right)\right)$ are used to compute the log-likelihood of the foreground and background, respectively. The logarithmic operation magnifies the difference and helps to increase the penalty for misclassification. $G\left(x\right)\log\left(\sigma \left(P\left(x\right)\right)\right)$ indicates the logarithmic value of the probability that the model predicts the pixel to be foreground when the true label *G*(*x*) is 1. By maximizing this value, the model predicts foreground pixels more accurately. $\left(1-G\left(x\right)\right)\log\left(1-\sigma \left(P\left(x\right)\right)\right)$ denotes the logarithmic value of the probability that the model predicts the pixel as background when the true label $G\left(x\right)$ is 0. By maximizing this value, the model will predict background pixels more accurately. Finally, the overall loss value is obtained by summing the losses for all pixels and averaging them to ensure that the loss is independent of image size.

(15) $$BCE\left(P,G\right)=-\frac{1}{\left|\Omega \right|}\sum_{x\in \Omega }\left[G\left(x\right)\log\left(\sigma \left(P\left(x\right)\right)\right)+\left(1-G\left(x\right)\right)\log\left(1-\sigma \left(P\left(x\right)\right)\right)\right]$$

where $Dice\left(P,G\right)$ is used to evaluate the similarity between the segmented areas predicted by the model and the actual segmented areas, especially robustness when dealing with unbalanced datasets. Its mathematical expression is:

(16) $$Dice\left(P,G\right)=1-\frac{2\sum_{x\in \Omega }\sigma \left(P\left(x\right)\right)\cdot G\left(x\right)+\xi }{\sum_{x\in \Omega }\sigma \left(P\left(x\right)\right)+\sum_{x\in \Omega }G\left(x\right)+\xi }$$

where ξ is the smoothing term, which is usually a very small constant used to avoid cases where the denominator is zero. This term is to ensure that valid loss values are obtained even when there is no overlap between the predicted and true labels, thus avoiding the problem of unstable values.

In summary, this paper proposes AFLM for the final objective function of model optimization, which is mathematically expressed as:

(17) L=α·BCE+β·Dice+γ·Boundary

where *α*, *β* and *γ* are the weight coefficients of each loss function, respectively, taking the values of: $\alpha =\frac{\sqrt{2}}{2}$, $\beta =1+\frac{\sqrt{2}}{2}$, $\gamma =1+\frac{\sqrt{2}}{2}$.

In this paper, we draw on the selection strategy for high-frequency features as well as low-frequency features in Scalelong [39], based on the principle that the composite loss function is the most robust loss function proposed by Ma et al. [40]. We then verify our ideas through a large number of comparative experiments and finally come up with the above weight ratios, which effectively take into account the advantages of different loss functions. Specifically, choosing $\alpha =\frac{\sqrt{2}}{2}$ to moderately reduce the impact of BCE loss is to ensure that the model does not pay excessive attention to the global classification accuracy while ignoring the processing of boundaries and details, which makes the model pay more attention to the processing of small goals rather than just optimizing the background. And larger values of *β* and *γ* are chosen to enhance the effect of Dice loss and boundary loss to improve the model’s segmentation performance on unbalanced datasets with small regions and complex boundaries. Increasing the β further emphasizes the model’s accurate prediction of small-target regions, which directly improves the Dice score to a certain extent; increasing *γ* emphasizes the accurate treatment of boundaries, which helps the model maintain high accuracy in complex segmentation scenarios.

With this weight adjustment, the model is able to achieve the best performance in dealing with small-target segmentation, boundary refinement, and overall segmentation consistency, thus improving the segmentation performance in general. Experiments show that the model with this loss function significantly improves the Dice coefficient on the nucleus Bowl 2018 dataset, validating the effectiveness and superiority of our method.

## 4. Experimental Results and Analysis

### 4.1. Datasets

Bowl 2018: The dataset used in this study is the Data Science Bowl 2018 nucleus segmentation dataset, hosted by Kaggle, with the aim of advancing research and applications in the field of nucleus segmentation. The Bowl 2018 dataset contains over 600 microscope images of cell nuclei, covering various tissue types, sample preparation methods, and staining techniques. Each image has been manually annotated by experts, precisely identifying every nucleus region. The images in the dataset involve a variety of nucleus types and shapes, including both single-nucleus and multi-nucleus scenarios.

MoNuSeg: The dataset of this challenge is obtained by carefully labeling the tissue images of tumor patients with several different organs and diagnosing them in multiple hospitals. The dataset was created by downloading a 40-fold enlarged H & E stained tissue image from the TCGA archive. H & E staining is a routine method to enhance the contrast of tissue sections, which is often used in tumor evaluation (grading, staging, etc.). The dataset has a total of 24 training images, 6 verification images, and 14 test data. Considering the diversity of nuclear morphology of multiple organs and multiple patients, and the richness of staining schemes adopted by multiple hospitals, the training dataset will help to develop robust and scalable nuclear segmentation techniques.

### 4.2. Implementations Setting

The experiments on the Bowl 2018 dataset were conducted on a V100 server using the Pytorch framework and Python 3.9. For data preprocessing, we applied random rotations, flips, scaling, and normalization, resizing all images to a format of 256 × 256. The AdamW optimizer was used with an initial learning rate set to 1 × 10^−3^, and a CosineAnnealingLR scheduler was employed, with a minimum learning rate of 1 × 10^−5^ and momentum set to 0.9. The batch size for the experiments was eight, and training was conducted for a total of 200 epochs.

The experiments on MoNuSeg dataset were conducted on a 3080 server using the Pytorch framework and Python 3.8. For data preprocessing, we adjusted all images to a 224 × 224 format. The Adam optimizer was used with an initial learning rate set to 1 × 10^−3^. The batch size for the experiments was four, and training was conducted for a total of 300 epochs.

### 4.3. Evaluation Metrics

To comprehensively evaluate the performance of the proposed nucleus segmentation method, we introduced several commonly used evaluation metrics. These metrics assess the consistency and differences between the segmentation results and the ground truth from different perspectives, providing a thorough performance analysis. Among them, the IoU and the Dice coefficient primarily focus on the coverage and overlap of the segmentation results, serving as key indicators of segmentation accuracy. Higher IoU and Dice values indicate greater overlap between the segmentation results and the ground truth, while lower values suggest significant discrepancies. Additionally, Precision, Recall, and Specificity are more focused on the precision of the segmentation results and error types. Precision measures how many of the predicted positive samples are true positives, Recall evaluates how many true positives are correctly identified by the model, and Specificity assesses the model’s ability to correctly predict negative samples.

By conducting a comprehensive analysis of these evaluation metrics, we can gain a full understanding of the performance of the nucleus segmentation method and identify specific areas for improving and optimizing the algorithm. These metrics not only provide a quantitative basis for algorithm performance but also offer scientific guidance for advancing research and development in the field of nucleus segmentation.

In this study, we conducted a comprehensive performance comparison across different models using the Bowl 2018 and MoNuSeg datasets. As shown in Table 1, we provide a detailed performance comparison on the Bowl 2018 dataset, highlighting the strengths and weaknesses of each model. Similarly, Table 2 presents the performance comparison on the MoNuSeg dataset, allowing for a broader understanding of model efficacy across different contexts.

**Figure 4:**
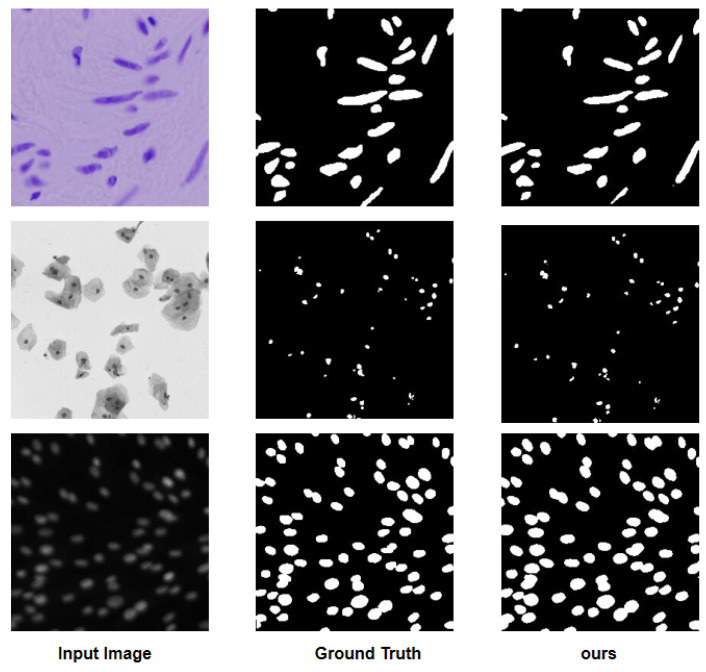
Visualized comparison diagram on the Bowl 2018.

**Figure 5:**
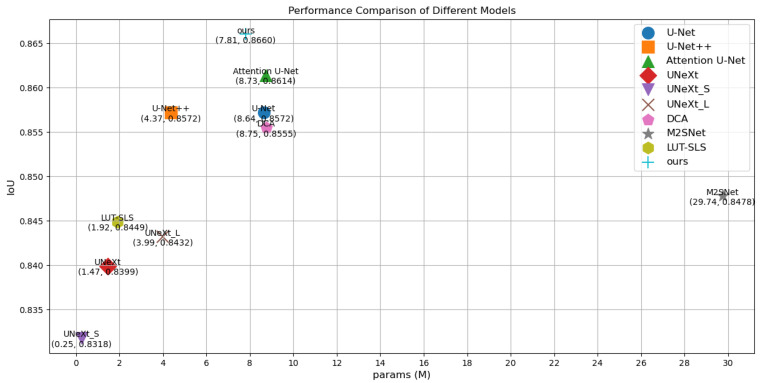
Comparison of params and IoU performance of different models on the Bowl 2018.

**Figure 6:**
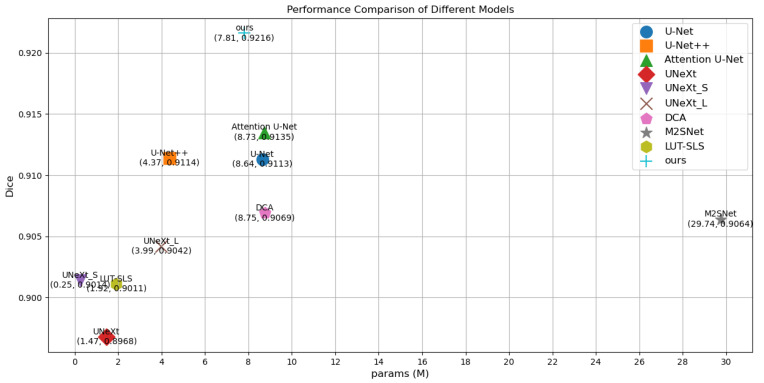
Comparison of params and Dice performance of different models on the Bowl 2018.

To visualize the results, Figure 4 illustrates a comparison diagram on the Bowl 2018, which highlights key differences in model performance. Furthermore, Figure 5 and Figure 6 delve into the relationship between model parameters and performance metrics, specifically focusing on Intersection over Union (IoU) and Dice coefficient performance, respectively. These visual comparisons not only emphasize the performance metrics but also facilitate a deeper understanding of how different models scale with parameter changes on the Bowl 2018 dataset.

## 5. Discussion

### 5.1. Ablation Experiments

This study, based on the Bowl 2018 nucleus dataset, conducted a series of ablation experiments to explore the performance and robustness of the proposed model under different conditions. During the experiments, each module was progressively introduced and analyzed to verify the impact of specific components on the model’s performance. The results of the specific ablation experiments are shown in Table 3.

First, all performance metrics show significant improvement after the introduction of WFEU. This indicates that the introduction of this module has a positive impact on the overall performance of the model. Secondly, the introduction of DSCOM successfully achieves a further improvement in the model performance. Finally, the introduction of AFLM resulted in an optimal overall performance of the model. This series of experimental results verifies the effectiveness of our method in model performance optimization and provides strong support for further research and application.

### 5.2. AFLM Weight Coefficient Analysis Experiment

The research under the AFLM module is based on the Bowl 2018 nuclear dataset. Through a series of ablation experiments with different proportions of weight coefficients, we aim to explore the effectiveness of the specific coefficients we proposed. It can be seen from Table 4 that the weight coefficient ratio selected in this paper performs well in each evaluation index, which confirms the effectiveness of this method in performance optimization.

## 6. Conclusions

This study proposes a novel nucleus segmentation method based on the U-Net architecture, integrating WFEU, DSCOM, and AFLM to significantly enhance the model’s performance in nucleus segmentation tasks. Experimental results show that the method outperforms existing state-of-the-art models across multiple key metrics on the Nucleus Bowl 2018 dataset, particularly excelling in handling small targets and complex boundaries. At the same time, the method effectively controls computational costs while ensuring high segmentation accuracy, demonstrating broad potential for practical applications.

These results not only validate the rationality and innovation of the module designs but also provide important references for model optimization in future medical image segmentation tasks. With the advancement of medical imaging technology and the expansion of dataset scales, the findings of this study are expected to play a key role in larger-scale, higher-resolution, and more complex image analysis tasks.

Future work will focus on further optimizing the model’s computational efficiency, allowing it to maintain high accuracy while achieving better performance on larger datasets and in real-time applications. Additionally, we will explore integrating this method with other advanced deep learning techniques, such as multi-modal data fusion and self-supervised learning, to further enhance the model’s generalization capabilities and applicability. Meanwhile, future research will also consider extending this method to other types of medical image analysis tasks, such as multi-organ segmentation and organ recognition, providing more effective technical support for precision medicine and clinical decision-making.

In conclusion, the field of nucleus segmentation still faces many challenges and opportunities. Through continued research and innovation, we aim to further improve the performance of nucleus segmentation and contribute to the advancement of medical imaging technology.

## Figures and Tables

**Table 1:** Performance comparison on the Bowl 2018. Bolded values in the table indicate the results with the best results.

Model	Params	GMac	IOU	Dice	Precision	Recall
U-Net (2015)	8.64	16.46	0.8572	0.9113	0.9204	0.9258
U-Net++ (2018)	4.36	31.03	0.8572	0.9114	0.9132	0.9332
Attention U-Net (2018)	8.73	16.74	0.8614	0.9135	0.9204	0.9306
UNeXt_S (2022)	0.25	0.10	0.8318	0.8968	0.8999	0.9164
UneXt (2022)	1.47	0.57	0.8399	0.9014	0.9004	0.9255
UNeXt_L (2022)	3.99	1.42	0.8432	0.9042	0.9031	0.9272
DCA (2023)	8.75	16.77	0.8555	0.9069	0.9144	0.9299
M2Snet (2023)	29.74	9.0	0.8478	0.9064	0.9072	0.9282
LUT-SLS (2024)	1.92	0.74	0.8449	0.9011	0.9011	0.9308
Ours	7.81	15.04	**0.8660**	**0.9216**	**0.9223**	**0.9342**

**Table 2:** Performance comparison on the MoNuSeg. Bolded values in the table indicate the results with the best results.

Model	Params	GMac	IOU	Dice
U-Net (2015)	8.64	12.60	0.6422	0.7731
U-Net++ (2018)	4.36	23.76	0.6402	0.7762
Attention U-Net (2018)	8.73	12.82	0.5228	0.6689
BiO-Net (2020)	14.97	27.44	0.6121	0.7483
UNeXt_S (2022)	0.25	0.08	0.5621	0.7107
UneXt (2022)	1.47	0.44	0.5659	0.7176
UNeXt_L (2022)	3.99	1.09	0.5897	0.7322
MALUNet (2022)	0.18	0.67	0.5069	0.6693
UCTransNet (2022)	66.43	32.93	0.6093	0.7497
ACC-Unet (2023)	16.77	45.25	0.6172	0.7519
LUT-SLS (2024)	2.06	0.57	0.5819	0.7285
Ours	7.81	11.52	**0.6538**	**0.7852**

**Table 3:** Ablation experiments results on the Bowl 2018.

Model	IOU	Dice	SPE
WFEU	0.8614	0.9138	0.9841
DSCOM	0.8655	0.9158	0.9857
AFLM	0.8660	0.9216	0.9859

**Table 4:** Different weight coefficient experiment results on the Bowl 2018. Bolded values in the table indicate the results with the best results.

Proportion of Weight Coefficient (BCE:Dice:Boundary)	IOU	Dice	Precision	Recall	SPE
1:1:0	0.8655	0.9158	0.9205	0.9352	0.9857
1:0:1	0.8623	0.9074	0.9245	0.9272	0.9866
0:1:1	0.8607	0.9226	0.9192	0.9311	0.9856
1:1:1	0.8647	0.9170	0.9216	0.9335	0.9858
0.5:1:1	0.8634	0.9188	0.9128	0.9411	0.9841
1:1:0.5	0.8621	0.9152	0.9144	0.9377	0.9844
0.5:1:0.5	0.8617	0.9177	0.9083	0.9436	0.9830
0.5:2:2	0.8635	0.9216	0.9175	0.9362	0.9851
$\frac{\sqrt{2}}{2}$:$1+\frac{\sqrt{2}}{2}$:$1+\frac{\sqrt{2}}{2}$	**0.8660**	**0.9216**	**0.9223**	**0.9342**	**0.9859**

## Data Availability

The data in this study are publicly available. The data were obtained from the Internet, but access is required due to privacy or ethical concerns. Access can be obtained by contacting the authors.
